# A Self-Determination Perspective in Healthcare: Leader–Member Exchange and Job Satisfaction in an Italian Sample

**DOI:** 10.3390/healthcare14060794

**Published:** 2026-03-20

**Authors:** Domenico Sanseverino, Alessandra Sacchi, Chiara Ghislieri

**Affiliations:** Department of Psychology, University of Turin, 10124 Turin, Italy; domenico.sanseverino@unito.it (D.S.); chiara.ghislieri@unito.it (C.G.)

**Keywords:** leader–member exchange, Self-Determination Theory, job satisfaction, hospital workers

## Abstract

**Background/Objectives**: Healthcare professionals operate in complex and demanding environments characterized by high workloads, emotional strain, and organizational pressures that can undermine well-being. According to Self-Determination Theory, the fulfillment of core psychological needs (autonomy, competence, and relatedness) leads to increased job satisfaction, a key indicator of occupational well-being. Additionally, leadership plays a central role in shaping needs-fulfilling environments. Drawing on Leader–Member Exchange Theory (LMX), which emphasizes that high-quality leader-follower relationships foster greater discretion, provide learning opportunities, and build constructive team interactions, this study aimed to examine whether supportive leadership is associated with job satisfaction through the mediation of autonomy, team task cohesion, and perceived training opportunities. **Methods**: Data were collected from a local health authority in Northern Italy through an anonymous online survey, completed by 697 healthcare professionals, including 546 non-medical healthcare staff (primarily nurses) and 151 physicians. Structural equation modeling with a robust maximum likelihood estimator was employed to test the mediation model, including professional role as a covariate. **Results**: Higher LMX was positively and directly associated with job satisfaction, through the partial mediation of autonomy, team cohesion, and training opportunities, all positively associated with satisfaction. Team task cohesion showed the strongest associations with both LMX and satisfaction. Physicians reported slightly higher levels of autonomy, training opportunities, and job satisfaction than non-medical professionals. **Conclusions**: The findings suggest that supportive leadership contributes to healthcare professionals’ job satisfaction both directly and indirectly by contributing to core needs fulfillment. Interventions that strengthen relational quality, promote team cohesion, and enhance professional development may help sustain well-being and adaptive functioning in high-demand healthcare environments.

## 1. Introduction

Healthcare contexts have long been characterized by high levels of complexity, work intensity, and continuous demands for professional updating [1,2,3]. In recent years, these enduring characteristics have been compounded by a broader healthcare workforce crisis, marked by declining job satisfaction, widespread fatigue and burnout risk, and elevated turnover intentions among healthcare professionals, causing a widespread personnel shortage [4,5]. Arguably, more than ever, healthcare organizations face the challenge of providing organizational resources and supportive practices that can foster more sustainable and satisfying work environments for healthcare professionals.

Research consistently shows that healthcare professionals’ job satisfaction, and thus their continuity within the profession, is closely linked to working conditions, leadership quality, and access to resources that support professional autonomy, competence, and collaboration [6,7,8,9].

Self-Determination Theory (SDT) [10] provides a robust framework for understanding how organizational factors may influence healthcare professionals’ motivation and well-being. According to SDT, the satisfaction of the basic psychological needs for autonomy, competence, and relatedness fosters optimal functioning, well-being, and positive work attitudes. Research has consistently shown the importance of high-quality relationships for healthcare workers, with several studies stressing the relationship between supportive leadership, job satisfaction and well-being [7,11,12]. Among the variety of operationalizations of supportive leadership, Leader–Member Exchange (LMX) theory highlights how high-quality leader–follower relationships provide access to resources, support, and opportunities that can fulfil core psychological needs [13,14,15].

Building on SDT and LMX, the present study examines how supportive leadership relates to job satisfaction among non-medical staff and physicians through three key work resources: job autonomy, training opportunities, and team task cohesion. These resources are conceptualized as contextual factors that support the satisfaction of the needs for autonomy, competence, and relatedness within healthcare work. Although both perspectives offer valuable insights into leadership and employee motivation, their integration has received limited attention in the literature, particularly within healthcare settings and in studies comparing different professional groups. By integrating motivational and relational perspectives, this study aims to contribute to the understanding of how leadership can foster sustainable well-being and satisfaction in healthcare organizations.

### 1.1. Leader–Member Exchange Theory and Job Satisfaction Among Healthcare Professionals

Supportive leadership has been widely recognized as a key factor for healthcare employees, with research consistently highlighting the positive association between high-quality leader–follower relationships and job satisfaction and well-being [7,11,12]. Leadership in healthcare has also been examined using multilevel approaches, highlighting how team-level leadership dynamics influence employee outcomes such as job satisfaction and well-being [16,17].

Specifically, Leader–Member Exchange theory [14] offers a relational perspective on leadership, highlighting that leaders develop differentiated relationships with each team member. These range from low-quality transactional interactions to high-quality exchanges characterized by trust, mutual respect, and reciprocal influence. In healthcare, high-quality relational leadership, including LMX, has been associated with multiple positive outcomes, such as reduced burnout and enhanced well-being [11,12,18], increased professional fulfilment, greater autonomy support and peer relationships [19], improved teamwork and safety climate [20], higher job satisfaction and reduced turnover intentions [7,9,21,22,23]. Among these variables, job satisfaction plays a central role, being one of the prime antecedents of reduced turnover intention and considering its well-known relationship with lower risk of burnout and improved patient care [24,25,26].

Job satisfaction refers to a positive evaluative judgment of one’s job, reflecting the extent to which work experiences meet individual needs, values, and expectations. In the healthcare context, job satisfaction plays a pivotal role in retaining healthcare workers, which is particularly critical given the current workforce shortages in this sector [4,27,28]. Job satisfaction among healthcare professionals appears to be shaped more by contextual and temporal factors than by professional role. Small differences have been observed across settings, with physicians reporting slightly higher satisfaction in emergency care and nurses in primary care, though overall evidence remains heterogeneous and inconclusive [29]. From an SDT perspective, job satisfaction can be understood as a distal outcome resulting from work environments that support the basic psychological needs for autonomy, competence, and relatedness [30,31].

In line with these considerations, high-quality leader–member exchanges are expected to be positively associated with job satisfaction among healthcare professionals.

**H1:**  *Supportive leadership is positively associated with job satisfaction.*

### 1.2. Leader–Member Exchange Theory, Self-Determination Theory and Job Satisfaction

Despite extensive research on leadership and need satisfaction, comparatively fewer studies have examined the intersection of leader–member exchange, need fulfillment, and job satisfaction in healthcare settings. A recent systematic review identified six main themes of organizational culture connected with job satisfaction in healthcare: communication with colleagues, supportive leadership, team cohesion, involvement in decision-making, employee recognition (including career growth and professional development), and autonomy [32]. Accordingly, the present study seeks to capture some of these themes and their relationships by framing job satisfaction within Self-Determination and Leader-Member Exchange theories.

Self-Determination Theory [10] provides a well-established theoretical lens for examining well-being in organizational settings [30,33,34,35], with specific declinations in healthcare contexts [36,37,38]. The core tenet of SDT is that human functioning and motivation depend on the extent to which three basic psychological needs are satisfied: autonomy, competence, and relatedness. Autonomy refers to the experience of acting with volition and psychological freedom, competence reflects the feeling of effectiveness in mastering tasks and challenges, and relatedness involves establishing and maintaining meaningful, caring connections with others [10]. When these needs are adequately fulfilled, individuals experience autonomous motivation, which supports well-being, engagement, and optimal functioning. In healthcare contexts, research has shown that need satisfaction is positively associated with job satisfaction and overall well-being among nurses and physicians [36,37,38].

Previous research has highlighted that high-quality leader–member exchanges can be understood as a social-contextual factor that facilitates the satisfaction of the three basic psychological needs [15,39]. In work contexts, supportive leadership can validate the self and thereby promote needs satisfaction [10,15,33,40,41]. By supporting employees and recognizing their value, leaders can facilitate access to need-supportive resources, such as an appropriate degree of autonomy, opportunities for professional development, and positive and constructive relationships with colleagues [15,42].

Despite the recognized role of high-quality leader–member exchanges in satisfying basic psychological needs, few studies have examined the integration of SDT and LMX [15,39,43], and none have focused specifically on the healthcare context. Therefore, the value of the present study lies in integrating these two theoretical perspectives within the healthcare context and in examining them across different professional groups, specifically physicians and non-medical staff.

### 1.3. Self-Determination Theory and Basic Psychological Needs at Work

In this section, we focus on three constructs, namely job autonomy, training opportunities, and team task cohesion, conceptualized as contextual resources that may support the satisfaction of the needs for autonomy, competence, and relatedness. As previously noted, SDT posits that the satisfaction of basic psychological needs within a given domain is shaped by the social context in which individuals operate [10,15,33,44]. Such contexts provide opportunities that enable individuals to experience autonomy, develop competence, and establish meaningful relationships with others. Within high-quality leader–member exchanges, employees may gain greater access to professional development opportunities, feel more valued as members of an interconnected team, and experience a stronger sense of personal initiative. These conditions can facilitate psychological need satisfaction [15] and, in turn, enhance job satisfaction [36,37,38]. Accordingly, in this study, job autonomy, training opportunities, and team task cohesion are examined as mediators of the relationship between supportive leadership and job satisfaction.

Starting with job autonomy, it is defined as the degree of freedom workers are granted to plan and organize their work, make decisions, and choose work methods [45,46]. In healthcare, autonomy for both nurses and physicians involves the ability to make independent clinical decisions and participate in organizational processes, although its scope varies across professional roles. Physicians generally have broader clinical autonomy and control over their workload, whereas nurses exercise autonomy mainly within patient care protocols and collaborative frameworks. While both groups can influence organizational decisions, physicians typically hold greater formal authority [47,48]. Although healthcare work is necessarily regulated, perceived job autonomy remains a fundamental aspect of professional practice. It provides the flexibility needed to deliver high-quality care and supports professionals’ sense of volition and autonomous motivation [49]. Consistent with this perspective, studies conducted across different countries have shown that job autonomy is positively associated with job satisfaction and quality of care [50,51,52,53,54]. Despite the structural constraints illustrated above, job autonomy in healthcare is rarely only determined by formal roles and regulations but is also shaped by relational processes. In particular, immediate supervisors are first in line in translating organizational culture into discretionary space for clinical judgment, participation, and decision-making. Generally speaking, followers in high-quality relationships, as defined by LMX, are granted a greater degree of trust and, as such, more discretion and participation in decision-making [55,56]. In the context of healthcare, empirical studies have shown that supportive and relational leadership styles are positively associated with nurses’ and physicians’ perceived job autonomy [23,57,58,59].

Alongside autonomy, person-centered leadership also strengthens social connectedness and collaborative engagement, both central themes for job satisfaction in healthcare. For the purpose of this study we focused on the latter, as task-based cohesion has been argued to be more impactful than its affective counterpart aspect in ensuring patient safety and optimal functioning and sustaining job satisfaction [5,60].

Task cohesion refers to the extent to which members of a team remain committed to shared goals and work together to successfully complete collective tasks. It reflects how well individuals coordinate their efforts, cooperate, and support each other in pursuit of common objectives, with strong alignment around team purpose and shared responsibility for performance outcomes [61,62]. On a more general note, within SDT, supportive social interactions are the riverbed to fulfill the need for relatedness [10]. In healthcare settings, task cohesion is an important driver of job satisfaction and retention, especially when professionals feel supported by peers during high-stress clinical tasks [60,63,64]. Given the importance of collaborative, multidisciplinary teamwork in healthcare, a relational approach to leadership has been found to be particularly effective in increasing team cohesion or its individual components. High-quality LMX has been linked to increased cooperative communication in healthcare, reduced role ambiguity and enhanced behavioral integration [65]. Furthermore, empowering and relational styles of leadership have been linked to better team functioning, role coordination, collaboration, and mutual support [66,67,68]. Taking into account evidence from other sectors, high-quality LMX relationships have been found to boost collaboration and knowledge sharing, with positive outcomes on team cohesion and overall performance [69].

While the delicate balance between autonomy and cohesion is arguably one of the most important factors for healthcare professionals, effectively exercising both independent decision-making and engaging in cooperative effort depends on sufficient competence. Access to training and professional development opportunities enables nurses and physicians to build the skills necessary to provide quality care and perform effectively in diverse teams, linking autonomy and relatedness closely to competence-supporting resources. These opportunities represent more than a means of skill acquisition: they signal organizational support, recognition, and investment in professionals’ long-term development [32]. Immediate supervisors often mediate access to learning opportunities, although they need the cultural and structural preconditions to provide spaces for continuous learning [70]. On this note, leaders engaged in high-quality exchange exhibit several behaviors aligned with personnel development: they show interest in staff ideas, facilitate access to information and training, and exchange informational feedback [15]. In healthcare contexts, supportive and person-centered leaders have been shown to enhance professionals’ access to learning and development opportunities, including non-technical skills, mentorship, and structured leadership preparation [71,72]. By providing opportunities for development, supportive leadership contributes to the fulfillment of the need for competence and, ultimately, to job satisfaction.

Building on these literature findings, the following hypotheses are proposed:

**H2:**  *Supportive leadership is positively associated with (a) job autonomy, (b) team task cohesion, and (c) training and professional development opportunities*

**H3:**  *(a) Job autonomy, (b) team task cohesion and (c) training opportunities are positively associated with job satisfaction and mediate the relationship between supportive leadership and job satisfaction.*

**Figure 1:**
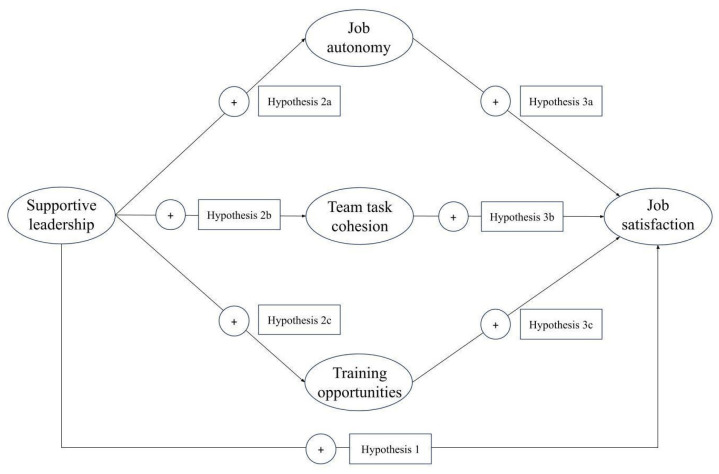
Theoretical model and study hypotheses.

The three hypotheses previously presented can be summarized in a model in which the satisfaction of the three basic psychological needs acts as a mediator in the relationship between supportive leadership and job satisfaction (Figure 1).

Building on this model, the present study aims to provide a consolidated contribution to the literature. First, it advances research by explicitly integrating Self-Determination Theory and Leader–Member Exchange theory within the healthcare context, an area that has received limited empirical attention despite the recognized connections between the two frameworks. Second, the study extends prior research by examining this integrated model across different professional groups within the same organizational setting. Specifically, by comparing physicians and non-medical staff, it offers novel insights into whether and how these theoretical mechanisms operate similarly or differently across professional roles, an issue that has been largely overlooked in existing studies.

## 2. Materials and Methods

### 2.1. Procedure

The study was conducted in a public health company in Northern Italy, with the data collection spanning from October 2023 to January 2024. The intervention was embedded in a tailored well-being assessment process, developed in close collaboration with organizational stakeholders to ensure alignment with the specific cultural and operational characteristics of the healthcare organization, and implemented with particular attention to participant involvement and the overall intervention process. The survey was conducted as part of the organization’s legally mandated workplace stress assessment. The inclusion of a neutral, third-party academic institution ensures that participants could answer anonymously and that the results would be shared only in aggregated form. For the quantitative phase of the project, which constitutes the focus of the present study, all employees in the organisation were sent a link to an online questionnaire hosted on Limesurvey (Version 3.27.22). The questionnaire was accompanied by a cover letter that outlined the objective of the research, compilation instructions, the voluntary and anonymous nature of the participation, the right to opt out of the study and finally the information concerning processing of personal and survey data. Only participants who gave informed consent to the collection, analysis, and publication of data were included in the analyses. Participation was voluntary and anonymous. The research did not include procedures that could harm participants’ psychological or social well-being, in compliance with the Declaration of Helsinki [73], and was conducted in compliance of the Italian data protection and privacy regulations (Law 196/2003) and the GDPR.

### 2.2. Participants

The final sample consisted of 697 healthcare professionals, including 546 non-medical staff members (primarily nurses and other allied health professionals) and 151 physicians, corresponding to an overall response rate of 48.1%. Compared to the reference population (1070 non-medical staff and 379 physicians), the sample composition was broadly consistent with the workforce distribution, although response rates were higher among non-medical staff (51.0%) than among physicians (39.8%). Most participants were females (73%). Due to privacy constraints, demographic information was collected using broad categorical ranges. Specifically, age and job seniority were assessed in aggregated classes, and no detailed information regarding contract type or specific job position was obtained. The largest proportion of participants fell within the 51–60 age group (32%), followed by those aged 41–50 years (28%), then the 31–40 range (24%), with workers younger than 30 years (9%) or older than 60 years (6%) representing the lowest share. With respect to job seniority, the most frequently reported categories were more than 20 years (46%), followed by 11–20 and 3–10 years of service, both endorsed by 22% of the sample. Table 1 presents a summary of the sample’s sociodemographic characteristics.

### 2.3. Data Analysis

We employed the Jamovi statistical software (version 2.3) [74] to conduct descriptive analyses, calculate Pearson’s correlation coefficients, and composite reliability through McDonald’s Omega. All measures presented acceptable values of skewness and kurtosis. Average variance extracted (AVE) was assessed in MPlus (Version 8) through confirmatory factory analysis, employing a Robust Maximum Likelihood estimator (MLR).

The hypothesized mediation model was tested using a structural equation model (SEM) in Mplus, employing an MLR estimator to account for potential deviations from multivariate normality and provide robust estimates for the confidence intervals of the indirect effects. Missing data was handled with a Full Information Maximum Likelihood approach. Supportive leadership, autonomy, team task cohesion, perceived training opportunities, and job satisfaction were specified as latent variables, each indicated by their respective observed items. Professional role (physician vs. non-medical healthcare professional) was included as an observed covariate predicting autonomy, team task cohesion, perceived training opportunities, and job satisfaction. Following the procedures outlined by Baron and Kenny [75], mediation effects were examined by testing the significance of indirect paths within the SEM framework. Indirect effects were tested using standardized estimates. The significance of total, total indirect, and specific indirect effects was evaluated using bias-corrected confidence intervals derived from the robust estimator. The proportion of explained variance (R^2^) was examined for each endogenous latent variable to assess the explanatory power of the model.

Model fit was evaluated based on multiple indices and criteria: the χ^2^ index, root mean square approximation error of approximation (RMSEA), comparative fit index (CFI) and residual standardized mean square deviation (SRMR). We considered the following cutoff values for acceptable model fit: >0.90 for CFI and TLI [76], <0.08 for both RMSEA [77] and SRMR [76] with associated confidence intervals. To assess the potential impact of common method variance, a Harman single-factor test was conducted by loading all observed items onto a single latent factor [78]. The one-factor model demonstrated poor fit to the data [χ^2^(253) = 5039.925, *p* < 0.001, RMSEA = 0.17, CFI = 0.49, TLI = 0.45, SRMR = 0.12], indicating that a single factor could not adequately account for the covariance among the measures. To examine whether the measurement model operated equivalently across professional roles, multigroup confirmatory factor analyses were conducted in Mplus8, testing configural, metric, and scalar models. In this study, professional group membership is not only a descriptive characteristic but is analytically used to test the invariance of the proposed model across physicians and non-medical staff. Due to the large sample size, the χ^2^ index was expected to be sensitive and was not interpreted. Instead, model comparison was based on changes in the fitness indices suggested by Chen [79], namely ΔCFI ≤ 0.01 with ΔRMSEA ≤ 0.015 and ΔSRMR ≤ 0.030 (metric) or 0.015 (scalar).

### 2.4. Measures

Supportive Leadership was assessed with five items from the Leader-Member Exchange Scale [14]. Participants indicated how frequently each statement applied to their relationship with their supervisor on a 5-point scale ranging from 1 (Never) to 5 (Always). An example item was “My supervisor uses his or her influence to help me solve my problems at work”. Skewness ranged between −0.47 and 0.25, and kurtosis between −1.22 and −0.81. Internal consistency was excellent and convergent validity was supported (McDonald’s ω = 0.94; AVE = 0.75).

Autonomy was assessed using three items [80] measuring the degree of discretion and independence experienced at work. Responses were provided on a 5-point frequency scale ranging from 1 (Never) to 5 (Always). An example item was “I have freedom to decide how I do my work”. Skewness ranged from 0.23 to 0.51, and kurtosis from −0.87 to −0.95; reliability and convergent validity were acceptable (McDonald’s ω = 0.75; AVE = 0.50).

Team task cohesion was measured using three items adapted from the Group Environment Questionnaire [61]. Participants rated the extent to which their team was united in accomplishing work tasks on a 5-point Likert scale. A representative item was “Our team is united in trying to reach its goals for performance”. Skewness ranged from −0.14 to 0.04, and kurtosis from −1.02 to −0.98. Reliability and convergent validity were excellent (McDonald’s ω = 0.95; AVE = 0.85).

Training opportunities were assessed with three items drawn from the six-item instrument employed in the study of Molino et al. [81], which covers information and training opportunities. Participants responded on a 5-point Likert scale indicating the adequacy of training and growth opportunities in their job. An example item was “I can learn new things and grow professionally”. Distribution was approximately normal, with skewness values between 0.23 and 0.28, and kurtosis between −0.85 and −0.81. Internal consistency and convergent validity were both strongly supported (McDonald’s ω = 0.95; AVE = 0.70).

Job Satisfaction was measured using nine items adapted from the COPSOQ II [82]. Participants were asked to consider their job overall and rate their satisfaction with various aspects of their work on a 5-point scale ranging from 1 (Very unsatisfied) to 5 (Very satisfied). An example item was “The way my skills are employed”. Skewness ranged from 0.17 to 0.60, and kurtosis from −0.98 to −0.63, with strong reliability and acceptable convergent validity (McDonald’s ω = 0.90; AVE = 0.50).

## 3. Results

### 3.1. Descriptive Statistics and Correlations

Table 2 presents the means, standard deviations, and correlations between the investigated variables. Supportive leadership was positively and moderately correlated with autonomy, team task cohesion, and perceived training opportunities. These associations are consistent with the hypothesized role of supportive leadership in fostering need-related and work-related resources.

Autonomy was positively associated with team task cohesion and training opportunities, while team task cohesion and training opportunities were also positively related. Overall, the pattern of correlations suggested related but distinct constructs, supporting their inclusion as separate latent variables in subsequent structural analyses, accounting for their correlation.

Professional role showed small but statistically significant correlations with autonomy and training opportunities, indicating that physicians tended to report slightly higher levels of these variables than non-medical healthcare professionals. Correlations between role and supportive leadership and between role and team task cohesion were non-significant.

Across participants, the perception of supportive leadership and team task cohesion was moderate, slightly below the middle point of the scale, while autonomy scores were comparatively higher.

### 3.2. Measurement Model and Invariance Testing

The baseline measurement model, including all latent constructs with no structural paths, demonstrated acceptable fit: χ^2^(220) = 942.38, *p* < 0.001; RMSEA = 0.069 [0.064, 0.073]; CFI = 0.922; TLI = 0.910; SRMR = 0.066.

Establishing measurement invariance is considered an important step when comparing latent constructs across groups in organizational and leadership research [83], as commonly done in leadership research e.g., in [84]. To examine measurement invariance across professional groups, a series of nested multi-group CFAs was conducted. The configural model showed an acceptable fit: χ^2^(440) = 1191.47, *p* < 0.001; RMSEA = 0.070 [0.065, 0.075]; CFI = 0.921; TLI = 0.909; SRMR = 0.068), indicating that the general factor structure was similar across groups. Constraining factor loadings across groups (metric invariance) yielded acceptable fit: χ^2^(458) = 1237.05, *p* < 0.001; RMSEA = 0.070 [0.065, 0.075]; CFI = 0.918; TLI = 0.910; SRMR = 0.072). The change in fit relative to the configural model was small (ΔCFI = 0.003, ΔRMSEA = 0.000, ΔSRMR = 0.004), well below the recommended threshold, supporting the equality of factor loadings. Constraining item intercepts (scalar invariance) still provided an acceptable fit: χ^2^(476) = 1343.57, *p* < 0.001; RMSEA = 0.072 [0.068, 0.078]; CFI = 0.909; TLI = 0.903; SRMR = 0.076. Changes relative to the metric model were within criteria (ΔCFI = 0.009, ΔRMSEA = 0.002, ΔSRMR = 0.004), confirming scalar invariance.

### 3.3. Hypothesis Testing

The hypothesized mediation model was tested using a SEM with MLR estimator. Model fit indices indicated acceptable fit: χ^2^(242) = 1069.86, *p* < 0.001, RMSEA = 0.070 [0.066, 0.074], CFI = 0.912, TLI = 0.900, and SRMR = 0.075. Supportive leadership indicators loaded strongly on the latent factor (λ = 0.74–0.94), autonomy items showed moderate to strong loadings (λ = 0.67–0.75), while team task cohesion exhibited very high loadings (λ = 0.90–0.95). Perceived training opportunities showed strong loadings in two out of three items (λ = 0.64–0.93). Finally, job satisfaction indicators loaded moderately to strongly on the latent construct (λ = 0.49–0.81).

**Figure 2:**
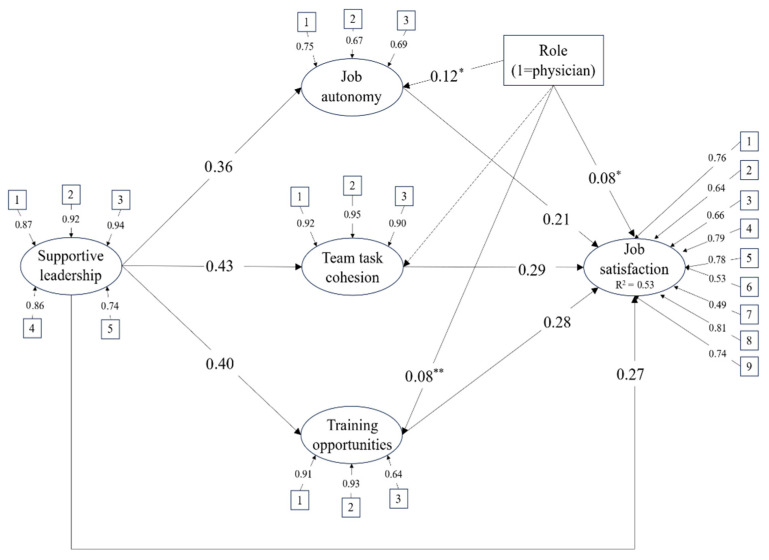
Results of the structural equation model (standardized path coefficients). Note: *p* < 0.001; * *p* < 0.01; ** *p* < 0.05. Dotted lines represent non-significant associations.

The diagram for the SEM is reported in Figure 2. Supportive leadership was positively and strongly associated with all three mediators; specifically, team task cohesion showed the strongest association, followed by training opportunities and finally by job autonomy. In turn, each mediator was positively related to job satisfaction, with similar coefficient sizes. Supportive leadership was also directly and positively related to job satisfaction, indicating partial mediation. Physicians reported higher levels of autonomy, perceived training opportunities and job satisfaction compared to non-medical healthcare professionals. No significant differences were found for team task cohesion. Standardized path coefficients with standard errors, *p*-values, and confidence intervals are reported in Table 3.

The total standardized effect of LMX on job satisfaction was large (β = 0.58), with the total indirect effect (β = 0.31), accounting for a meaningful proportion of the overall association. Examination of specific indirect effects revealed that all three mediators contributed significantly to the relationship. Confidence intervals for all indirect effects did not include zero, confirming their statistical significance (Table 4).

Overall, team task cohesion was the variable showing the strongest association with both supportive leadership and job satisfaction, although training opportunities were a close second. The model accounted for 14.2% of the variance in autonomy, 18.1% in team task cohesion, and 16.4% in perceived training opportunities. Finally, the model explained 52.6% of the variance in job satisfaction.

## 4. Discussion

The findings of this study generally support the proposed model. In the following sections, the results are discussed in detail in relation to the specific hypotheses.

High-quality leader–member exchanges were found to be directly associated with job satisfaction in the healthcare context, thereby supporting Hypothesis 1, which posited a positive relationship between supportive leadership and job satisfaction. Job satisfaction is a key determinant of healthcare professionals’ well-being and care quality, as it represents one of the main antecedents of reduced turnover intentions, lower burnout risk, and improved patient care [24,25,26]. At the same time, evidence of its association with supportive leadership [21,22] underscores the importance, within healthcare settings, of investing in the development of high-quality leader–member exchanges.

Hypothesis 2, which posited that supportive leadership is positively associated with job autonomy, team task cohesion, and training and professional development opportunities, was supported. Overall, the findings reinforce the role of leadership in promoting the satisfaction of employees’ psychological needs, particularly through high-quality leader–member relationships, as conceptualized by LMX theory [13,14,15]. Although the relationship between leadership and basic psychological needs has received some attention within the SDT framework, empirical evidence remains limited. The present study contributes to this line of research by examining these dynamics within the Italian healthcare sector. Specifically, the study considered job autonomy, opportunities for professional development, and team task cohesion as contextual facilitators associated with the satisfaction of autonomy, competence, and relatedness, respectively. Consistent with previous evidence showing that high-quality LMX relationships grant followers greater access to support, autonomy, and developmental resources [13,42,55], these associations were confirmed in the healthcare context, particularly among nurses and physicians.

Among the three SDT-related variables considered, supportive leadership showed the strongest association with team task cohesion. This finding is consistent with prior evidence highlighting the capacity of high-quality LMX relationships to foster collaboration and strengthen team cohesion. This effect appears particularly salient in healthcare settings, where supportive leader–follower relationships have been shown to enhance cooperative communication, team functioning, role coordination, collaboration, and mutual support [65,66,67,68].

The second strongest relationship, although very close in magnitude to the first, concerned opportunities for training and professional development. Leadership likely plays a critical role in this domain because immediate supervisors often act as gatekeepers to learning and developmental resources [70]. Moreover, leaders engaged in high-quality exchanges tend to enact behaviors conducive to employee development, such as showing interest in staff ideas, facilitating access to information and training, and providing informational feedback [15].

The association between supportive leadership and job autonomy, while positive and comparable in strength to the other relationships, emerged as the weakest of the three. This result should be interpreted in light of the specific characteristics of healthcare work. On the one hand, job autonomy in healthcare is influenced by relational processes [55,56], and supportive leadership has been shown to be positively associated with perceived autonomy among nurses and physicians [57,58,59]. On the other hand, the effect of leadership on job autonomy may be partially constrained by structural and role-specific factors, such as formal authority, with physicians generally enjoying broader autonomy than nurses within healthcare organizations [47,48]. Supportive leadership thus confirmed its positive association with the satisfaction of the psychological needs postulated by SDT in this context.

Hypothesis 3 was supported, indicating that job autonomy, team task cohesion, and training and professional development opportunities are positively associated with job satisfaction and mediate the relationship between supportive leadership and job satisfaction. However, the persistence of a direct association between supportive leadership and job satisfaction suggests that this mediation is partial rather than full. In line with SDT [10], the fulfillment of psychological needs, which in this study was connected with high-quality leader–member relationships, was found to be positively associated with job satisfaction among nurses and physicians. Moreover, the relative strength of these associations mirrors the pattern observed for Hypothesis 2. Indeed, the strongest association with job satisfaction emerged for team task cohesion, which reflects the need for relatedness as conceptualized within Self-Determination Theory. As previous research highlighted, task cohesion in healthcare settings plays a critical role in enhancing job satisfaction and reducing turnover intentions [60,63,64].

In conclusion, the findings provide support for the hypothesized model, suggesting that SDT represents a useful framework for understanding the dynamics that promote job satisfaction in the healthcare sector. Moreover, the results indicate that LMX theory offers a valuable perspective for explaining how high-quality leader–member relationships contribute to the satisfaction of psychological needs within this context. In the Italian context these theoretical frameworks have been applied only marginally and mostly separately in healthcare research, with studies focusing either on LMX e.g., in [85,86] or on SDT e.g., in [87,88], but rarely considering them together. Our findings, in line with previous studies, underscore the importance of examining the healthcare environment through these lenses, while also offering an attempt to integrate SDT and LMX to better capture the dynamics specific to the Italian setting.

Moreover, the results also revealed significant differences between physicians and nurses in terms of job autonomy, access to training opportunities, and job satisfaction. These differences can be attributed to the distinct roles of the two groups: although nurses exercise autonomy and participate in organizational decision-making, physicians generally hold greater formal authority and autonomy [47,48]. These role differences can also affect access to training and professional development opportunities. As previously discussed, the fulfillment of autonomy and competence needs fosters job satisfaction, which accordingly was higher among physicians, although such variations between roles may also reflect contextual and temporal factors rather than intrinsic professional differences [29]. These differences raise the possibility that professional role could moderate the relationships between autonomy, team task cohesion, training opportunities, and job satisfaction. Specifically, given that physicians generally enjoy more clinical autonomy and access to developmental opportunities and resources, which arguably are both aspects that are central to their professional identity, the strength of the associations between these mediators and job satisfaction may be greater for them compared to non-medical staff.

Finally, when interpreting the results of this study, it is useful to consider the main contextual factors related to the healthcare system, particularly within the Italian context.

First, it is important to acknowledge that the data were collected starting in 2023, not long after the outbreak of the global COVID-19 pandemic. Several studies have highlighted that the pandemic crisis has influenced relational and motivational factors among healthcare workers, such as teamwork climate, leadership engagement, improvement readiness, autonomy, and trust within the team [89,90]. Therefore, it is possible that, in the present study as well, perceptions of the examined variables and their relationships may have been influenced by this broader contextual condition.

Second, it is relevant to briefly outline the principal features of the Italian healthcare system [91]. Although the system is currently undergoing transformation, with a gradual increase in the presence of private healthcare providers, it remains predominantly public. Healthcare services are provided free of charge through facilities managed at the regional level, while overall coordination and policy direction are maintained at the national level. Public health policies are defined by the Ministry of Health and implemented by regional authorities. Despite the existence of a national framework, some regional differences persist. These differences are partly associated with variations in socioeconomic conditions and lifestyles across regions, as well as with the longstanding economic and social imbalance between Northern and Southern Italy. Nevertheless, Italian healthcare facilities share several structural and organizational features due to their integration within the same national system [91]. Although ongoing efforts aim to standardize healthcare services at the national level, including the organization of specific healthcare services, regional variations remain evident [92]. Therefore, the results discussed in this study should be interpreted in light of the fact that, although all Italian regions share common characteristics within the healthcare system, certain regional specificities exist. The generalizability of the findings could be strengthened through replications in other contexts.

### 4.1. Limitations and Future Directions

Multiple limitations should be considered when interpreting the results. First, the cross-sectional design does not allow us to draw causal inferences regarding the investigated relationships. Although the model was theoretically grounded and supported by previous empirical findings, the temporal ordering of variables cannot be established. It is therefore possible that a reciprocal relationship exists, where higher job satisfaction also influences employees’ perception of leadership quality and the availability and salience of work-related resources. For example, employees who experience greater satisfaction may evaluate leadership relationships more positively or may be more inclined to engage in proactive behaviors that strengthen leader–member exchanges over time, which would influence the reciprocal exchange of resources. Longitudinal and, possibly, multi-level studies would provide a more rigorous picture of these processes over time.

Second, all variables were assessed using self-report measures, which may increase the risk of social desirability and common method variance. Although the Harman single-factor test suggested that common method variance was unlikely to account for the observed associations, the procedure is a conservative diagnostic rather than definitive proof. Employees could have adopted self-presentation strategies, leading to overly cautious or optimistic reporting. However, considering the relatively low scores in our study variables, with the exception of autonomy, this case seems unlikely. This does not rule out the possibility of cognitive consistency in answers: participants rating their relationship with the leaders as more positive could have later reported higher levels of resources to maintain an internal consistent narrative. Future research would benefit from adopting multi-source designs, including measures such as supervisor-rated leadership or objective indicators of training and professional development, to reduce risks of recall, perceptual, and common rater biases.

Third, the study was conducted in a single context in Northern Italy, which represents a limitation. As previously noted, although a national health system guides public health policies and shapes organizational functioning, Italy exhibits significant differences between the North and the South, as well as additional specificities linked to regional implementation [91,92]. Considering how leadership practices, team dynamics, and access to developmental resources are deeply influenced by organizational, economical, and cultural factors that vary across healthcare systems and national contexts, caution is warranted when extending these results to other healthcare settings. Replication of the model across diverse healthcare settings would strengthen its robustness and generalizability, particularly by examining how healthcare governance and organizational culture affect hierarchical structures and levels of autonomy in resource allocation—elements that may impact the study’s core variables.

Fourth, a limitation of the present study is that the majority of respondents were female. This gender composition reflects the workforce realities in the study context but may have influenced the patterns of leadership perception reported.

Fifth, although the overall response rate was satisfactory and the sample composition broadly reflected the population distribution, physicians showed a considerably lower response rate compared to non-medical healthcare professionals. While the role was included in the analyses, differential response rates may still have influenced the observed role-related differences in the study variable.

Sixth, leadership perceptions and team cohesion may be shaped by hierarchical and departmental contexts, which our independent-observation approach does not capture. Multilevel modeling would allow future research to more accurately reflect these nested influences.

Finally, due to privacy constraints, several important demographic and occupational variables were either not collected or reported in aggregated categories. This limited the possibility of examining how contextual or employment-related factors might interact with the perception of supportive leadership and job resources.

### 4.2. Theoretical Implications

The quality of life of healthcare workers is closely linked to job satisfaction [29,93,94,95], which in turn represents a crucial psychological experience, a juncture of great importance in the work process, in a dynamic that has significant effects on the quality of service and therefore on the care that patients receive [93]. In this complex system of connections, understanding the psychosocial processes that influence satisfaction within a multidimensional framework is particularly relevant. Through this study, we have highlighted that SDT [10] can offer a useful framework in explaining job satisfaction from both a theoretically comprehensive and a highly practical perspective [30,33,34,35]. The study confirmed the relationship of supportive leadership with job satisfaction maintenance, which seems also to pass through the three constructs of job autonomy, team-task cohesion and perceived training opportunities, each of which is linked to one of the three core needs proposed by SDT.

Importantly, only a limited number of studies have attempted to integrate SDT and LMX, and even fewer have done so in healthcare settings. As noted in prior research combining these two frameworks, LMX has primarily been examined through the lens of Social Exchange Theory, with comparatively little attention to its links with employees’ internal psychological processes and individual outcomes [15,39,43]. The present study contributes to addressing this gap by offering an integrated perspective that connects leadership quality, psychological need satisfaction, and job-related outcomes. Considering the Italian context, where the application of SDT and LMX in healthcare has been limited, the findings highlight the value of combining these frameworks to capture the dynamics of professional motivation and well-being of healthcare professionals. Although LMX is inherently relational in nature (something reflected in its relatively stronger association with team task cohesion), the results also showed that all three resources, conceptualized as contextual facilitators of core needs fulfillment, displayed relationships of comparable strength with both LMX and job satisfaction.

Finally, the observed differences between professional roles (physicians vs. non-medical professionals) suggest that theoretical models should consider role-specific boundary conditions, such as the salience of autonomy, access to developmental opportunities, and relational resources may vary across occupational groups, potentially moderating the relationships proposed by SDT and LMX. Taken together, these findings offer insights into possible extensions of prior theory by providing an integrated lens for understanding leadership, need satisfaction, and job outcomes in healthcare, and by emphasizing the importance of contextual and role-specific factors in shaping these dynamics.

### 4.3. Practical Implications

This study highlights the role played by positive leader–member exchanges in supporting employee job satisfaction through the satisfaction of basic psychological needs. Therefore, the practical implications of the study reinforce the importance, particularly in healthcare settings, of promoting organizational contexts characterized by supportive leadership. This can be achieved by placing greater emphasis on soft skills during the recruitment and selection of individuals who will occupy supervisory roles, as well as through continuous leadership development initiatives, both in classroom-based programs and in individualized formats that support reflective work on experience (e.g., coaching and counseling) [96,97].

In addition, the three dimensions of job autonomy, team task cohesion, and training opportunities—linked to the satisfaction of the basic needs for autonomy, relatedness, and competence—proved to be relevant for job satisfaction and can be further strengthened through targeted organizational investment. The definition of areas of decision-making and operational autonomy can represent a structural feature of work organization, within the limits allowed by the specific role and type of activity involved. Team task cohesion can be further developed through team training initiatives, including interactive classroom activities, team coaching practices, or intensive training experiences such as outdoor programs. Finally, training initiatives should generally be designed as responses to real organizational challenges and to employees’ developmental needs, thereby fostering both effectiveness and employability.

## 5. Conclusions

Job satisfaction represents a central psychological experience in healthcare work, with important implications for workers’ well-being and for the quality of services delivered to patients. Understanding the psychosocial processes associated with job satisfaction within a multidimensional framework is therefore particularly relevant.

The present study shows that SDT provides a useful framework for interpreting job satisfaction in healthcare settings. In particular, the findings highlight the relevance of supportive leadership and its association with job satisfaction through three work-related dimensions: job autonomy, team-task cohesion, and perceived training opportunities, each linked to the satisfaction of one of the basic psychological needs proposed by the SDT. The study also contributes to the literature by examining these mediating mechanisms simultaneously within a public healthcare context and by offering comparative insights between physicians and non-medical healthcare professionals.

However, these findings should be interpreted in light of several limitations, including the cross-sectional design, the reliance on self-reported measures, and the fact that the study was conducted within a single public healthcare organization. These factors limit causal interpretation and the generalizability of the results, highlighting the need for further research using longitudinal, multi-source, and multi-level approaches across diverse healthcare contexts.

## Figures and Tables

**Table 1:** Study sample characteristics.

Sample Characteristics	Non-Medical Staff (*n* = 546)		Physicians (*n* = 151)		Total Sample (*n* = 697)	
	*n*	*%*	*n*	*%*	*n*	*%*
Gender						
Female	433	79.3%	79	52.3%	512	73.5%
Male	78	14.3%	69	45.7%	147	21.1%
Undisclosed	35	6.4%	3	2.0%	38	5.4%
Age						
<30	62	11.4%	0	0.0%	62	8.9%
31–40	129	23.6%	38	25.2%	167	24.0%
41–50	158	28.9%	40	26.5%	198	28.4%
51–60	177	32.4%	49	32.5%	226	32.4%
>60	20	3.7%	24	15.9%	44	6.3%
Job seniority						
<3	54	9.9%	19	12.6%	73	10.5%
3–10	110	20.1%	40	26.5%	150	21.5%
11–20	112	20.5%	44	29.1%	156	22.4%
>20	270	49.5%	48	31.8%	318	45.6%

**Table 2:** Means (*M*), Standard Deviations (*SD*), and Pearson correlations of the study variables.

Variable	1	2	3	4	5
1. Supportive leadership	—				
2. Autonomy	0.32 ***	—			
3. Team task cohesion	0.39 ***	0.24 ***	—		
4. Training opportunities	0.39 ***	0.28 ***	0.29 ***	—	
5. Professional role (1 = Physicians)	0.03	0.11 **	−0.02	0.09 *	—
*M*	2.85	3.60	2.94	2.54	
*SD*	1.21	0.82	1.18	1.03	

Note: *** *p* < 0.001; ** *p* < 0.01; * *p* < 0.05.

**Table 3:** Standardized path coefficients, standard errors, and 95% confidence intervals.

Dependent Variable	Predictor	β	S.E.	*p*	95% CI	R^2^
Autonomy	Supportive leadership	0.36	0.056	<0.001	[0.25, 0.47]	0.142
	Role	0.12	0.043	0.005	[0.04, 0.20]	
Team task cohesion	Supportive leadership	0.43	0.037	<0.001	[0.35, 0.50]	0.181
	Role	−0.03	0.037	0.354	[−0.11, 0.04]	
Training opportunities	Supportive leadership	0.40	0.037	<0.001	[0.32, 0.47]	0.164
	Role	0.08	0.036	0.025	[0.01, 0.15]	
Job satisfaction	Supportive leadership	0.27	0.041	<0.001	[0.19, 0.35]	0.526
	Autonomy	0.21	0.045	<0.001	[0.13, 0.30]	
	Team task cohesion	0.29	0.039	<0.001	[0.21, 0.36]	
	Training opportunities	0.28	0.041	<0.001	[0.20, 0.36]	
	Role	0.08	0.03	0.005	[0.03, 0.14]	

Note: 95% S.E. = Standard error; CI = 95% confidence interval; R^2^ = proportion of variance explained.

**Table 4:** Standardized indirect effects with 95% confidence intervals.

Indirect Effect (X = Supportive Leadership)	β	S.E.	*p*	95% CI
Autonomy → Job satisfaction	0.08	0.020	<0.001	[0.03, 0.12]
Team task cohesion → Job satisfaction	0.12	0.024	<0.001	[0.08, 0.16]
Training opportunities → Job satisfaction	0.11	0.023	<0.001	[0.07, 0.15]
Total indirect	0.31	0.035	<0.001	[0.25, 0.37]

## Data Availability

The data presented in this study are available on request from the corresponding author due to privacy restrictions.

## Abbreviations

AVE	Average Variance Extracted
CFI	Comparative Fit Index
CI	Confidence Interval
LMX	Leader–Member Exchange
M	Mean
MLR	Maximum Likelihood Estimation with Robust Standard Errors
R^2^	Proportion of Variance Explained
RMSEA	Root Mean Square Error of Approximation
SD	Standard Deviation
SDT	Self-determination theory
SRMR	Standardized Root Mean Square Residual
TLI	Tucker–Lewis Index
β	Standardized Regression Coefficient
λ	Factor Loading
χ^2^	Chi-Square Test of Model Fit
